# Prognostic Impact of Nutritional Status in Patients with Candidemia

**DOI:** 10.3390/nu18060936

**Published:** 2026-03-16

**Authors:** Nobuhiro Asai, Wataru Ohashi, Yuichi Shibata, Daisuke Sakanashi, Hideo Kato, Mao Hagihara, Hiroshige Mikamo

**Affiliations:** 1 Department of Clinical Infectious Diseases, Aichi Medical University, Nagakute 480-1195, Aichi, Japan; asai.nobuhiro.039@aichi-med-u.ac.jp (N.A.); shibata.yuuichi.414@mail.aichi-med-u.ac.jp (Y.S.); katou.hideo.233@mail.aichi-med-u.ac.jp (H.K.); hagimao@aichi-med-u.ac.jp (M.H.); 2 Department of Infectious Control Diseases, Aichi Medical University, Nagakute 480-1195, Aichi, Japan; sakanashi.daisuke.617@mail.aichi-med-u.ac.jp; 3 Division of Biostatistics, Clinical Research Center, Aichi Medical University, Nagakute 480-1195, Aichi, Japan; wohashi@aichi-med-u.ac.jp; 4 Department of Pharmacy, Mie University Hospital, Tsu 514-8507, Mie, Japan; 5 Department of Molecular Epidemiology and Biomedical Sciences, Aichi Medical University Hospital, Nagakute 480-1195, Aichi, Japan

**Keywords:** candidemia, CONUT score, GLIM criteria, malnutrition, EQUAR Candida score

## Abstract

**Background:** Candidemia remains a life-threatening infection with high mortality despite advances in antifungal therapy. Malnutrition has been suggested as an important contributor to poor outcomes; however, the prognostic value of different nutritional assessment tools in candidemia has not been fully clarified. This study aimed to evaluate the impact of nutritional status on prognosis in patients with candidemia and to identify the most reliable nutritional assessment tool. **Method:** We conducted a retrospective cohort study of adult patients diagnosed with candidemia at a tertiary teaching hospital in Japan between 2014 and 2024. A total of 170 patients were included, with a mean age of 73 years. Nutritional status was assessed using the Global Leadership Initiative on Malnutrition (GLIM) criteria and Controlling Nutritional Status (CONUT) score. The primary outcome was 30-day mortality. **Results:** According to the GLIM criteria, 72% of patients were classified as malnourished. The 30-day and in-hospital mortality rates were 35% and 44%, respectively. The CONUT score demonstrated good prognostic performance for 30-day mortality (Area under the Receiver operating characteristic 0.723, *p* < 0.001), with an optimal cut-off value of 12. Patients with higher CONUT scores had significantly shorter overall survival (*Log-rank* test *p* < 0.001). In multivariate analysis, a CONUT score ≥ 12, SOFA score ≥ 5, and the European Confederation of Medical Mycology Quality of Clinical Candidemia Management (EQUAL) Candida score ≥ 10 were independent predictors of 30-day mortality, whereas malnutrition defined by the GLIM criteria was not. **Conclusions:** The CONUT score is a useful prognostic indicator for short-term mortality in patients with candidemia, outperforming the GLIM criteria.

## 1. Introduction

Despite significant advances in diagnostic methods and antifungal therapies, candidemia remains a major clinical challenge due to its persistently high mortality rate of 30–60% [1,2,3]. Candidemia, a bloodstream infection caused by Candida species, typically arises when the normal mucosal or skin barriers are breached, allowing the translocation of Candida from colonized sites into the bloodstream. This process is often facilitated by factors such as the presence of central venous catheters, use of broad-spectrum antibiotics that disrupt the normal microbiota, immunosuppression, surgical procedures, and prolonged hospitalization, particularly in intensive care units [4].

Under normal conditions, host defenses—including intact epithelial barriers, neutrophil function, and innate immune responses—are critical in preventing fungal invasion. However, when these defenses are compromised, Candida can enter the bloodstream and disseminate to various organs, leading to life-threatening systemic infection [4]. Malnutrition has been increasingly recognized as a significant risk factor that contributes to the development and progression of candidemia [5,6]. Protein–energy malnutrition impairs both innate and adaptive immunity, reducing phagocytic activity, cytokine production, and mucosal integrity. Additionally, low serum albumin levels, which are often used as markers of nutritional status, are associated with increased vascular permeability and poor wound healing, further facilitating microbial invasion and translocation. Furthermore, malnourished patients—especially those with chronic illnesses or frailty—often require invasive medical interventions such as feeding tubes or central venous catheters, which themselves are known risk factors for candidemia. Therefore, malnutrition may play both a direct and indirect role in the pathogenesis of candidemia by weakening host defenses and increasing exposure to procedural risks. Recently, several nutritional tools have been used to assess patients’ conditions in general medical settings, such as Global Leadership Initiative on Malnutrition (GLIM) criteria [7], Controlled Nutritional Status (CONUT) score [8], Prognostic Nutritional Index (PNI) [9], and Geriatric Nutritional Risk Index (GNRI) [10]. However, it remains unclear whether these nutritional tools can forecast outcomes in candidemia. While factors such as advanced age, immunosuppression, Acute Physiology, Age, Chronic Health Evaluation II (APACHE II), and Sequential Organ Failure Assessment (SOFA) scores have been well established as predictors of poor outcomes, the prognostic significance of malnutrition in candidemia has yet to be fully elucidated. Therefore, we conducted this retrospective study to investigate whether nutritional assessment tools can predict outcomes in candidemia patients, and to determine which tools most accurately reflect prognosis.

## 2. Materials and Methods

### 2.1. Study Design and Patient Enrollment

This retrospective study was conducted at our institute, a tertiary teaching hospital with 800 beds located in a rural area of Aichi Prefecture, Japan. The objective was to identify which nutritional assessment tools best predict the prognosis of candidemia between 2014 and 2024.

We included patients aged ≥16 years who were diagnosed with candidemia at our institution. Candidemia was defined as the presence of at least one positive blood culture for *Candida* species, in accordance with previous studies [11]. Patients were excluded if clinical data were insufficient or if follow-up was not possible due to transfer to another medical facility.

This study was approved by the Institutional Review Board of Aichi Medical University Hospital (Approval No. 2025-040).

### 2.2. Patient Characteristics and Disease Severity

A total of 170 patients with candidemia were enrolled in the study. We evaluated patients’ characteristics including age, sex, underlying diseases, and nutritional status. Clinical outcomes such as initial antifungal therapy, duration of hospitalization, and treatment regimens were also assessed.

Disease severity was evaluated using the Systemic Inflammatory Response Syndrome (SIRS), quick Sequential Organ Failure Assessment (qSOFA), and SOFA scores. Adherence to the candidemia management bundle was assessed using the European Confederation of Medical Mycology Quality of Clinical Candidemia Management (EQUAL) Candida score [11,12]. The EQUAL Candida Score is a quality-of-care scoring system developed by the European Confederation of Medical Mycology. It measures adherence to guideline-recommended management of candidemia and invasive candidiasis. The score assigns points to key diagnostic, therapeutic, and follow-up interventions. In patients with a central venous catheter (CVC), the maximum score is 22 points, whereas in patients without a CVC, the maximum score is 19 points, because catheter removal cannot be assessed. Higher scores indicate better adherence to recommended management strategies. The score is primarily used for quality assessment and antifungal stewardship rather than for individual risk prediction [11,12].

Nutritional status was evaluated based on the GLIM criteria [7], CONUT score [8], PNI [9], and Geriatric Nutritional Risk Index (GNRI). A comparison of these nutritional indices is presented in Supplemental Table S1. Underlying comorbidities were assessed using the Charlson Comorbidity Index (CCI). Disseminated intravascular coagulation (DIC) was diagnosed using the Japanese Association for Acute Medicine (JAAM) DIC criteria [13]. Septic shock was defined as a systolic blood pressure <90 mmHg or the requirement for vasopressor support, in line with previous definitions [11].

### 2.3. Data Collection

We collected demographic and clinical data, including age, sex, underlying conditions, use of immunosuppressive agents, known risk factors for candidemia [14,15,16,17], and the infection source. Laboratory data were obtained on the day a positive blood culture was collected. Additionally, 30-day mortality, in-hospital mortality, and the date of last follow-up were recorded to calculate overall survival (OS). The EQUAL Candida score was assessed for each patient throughout the study period.

### 2.4. Microbiological Evaluation

*Candida* species were identified using the VITEK-MS system (bioMérieux, Marcy-l’Étoile, France). Antifungal susceptibility testing for amphotericin B, caspofungin, fluconazole, itraconazole, and voriconazole was conducted using the VITEK-2 AST-YS07 card (bioMérieux). Minimum inhibitory concentrations (MICs) were interpreted according to Clinical and Laboratory Standards Institute (CLSI) guidelines [18,19], using species-specific breakpoints for caspofungin (CPFG), fluconazole (FLCZ), itraconazole (ITCZ; for *C. albicans*), and voriconazole (VRCZ) [19]. Susceptibility to amphotericin B (AMB) and liposomal amphotericin B (L-AMB) was interpreted based on species-specific breakpoints established by the European Committee on Antimicrobial Susceptibility Testing (EUCAST), in accordance with prior studies [18].

### 2.5. Statistical Analyses

Categorical variables are presented as percentages, and continuous variables as mean ± standard deviation (SD). The Chi-square or Fisher’s exact test (two-tailed) was used for categorical variables, while the unpaired Student’s *t*-test or the Mann–Whitney *U* test was applied for continuous variables. Variables with a *p*-value < 0.10 in univariate analysis were included in multivariate models. Multivariate analysis was adjusted for potential confounders previously reported [14,15,16,17]. All statistical analyses were performed using SPSS version 26 for Windows (SPSS Inc., Chicago, IL, USA). Kaplan–Meier survival curves were generated using GraphPad Prism version 10. Overall survival (OS) was defined as the time from diagnosis to death from any cause. Comparisons of OS between groups were conducted using the log-rank test. A *p*-value < 0.05 was considered statistically significant.

## 3. Results

Table 1 shows patient characteristics and clinical outcomes. The mean age was 73 years old [(±standard deviation (SD) 13.7]. They were 108 males (64%) and 62 females (36%). The most frequent infection site was CRBSI in 91 (53%), followed by unknown in 71 (42%). As for nutritional status, the mean body mass index (BMI) and CONUT score were 19.2 (±3.9) and 9.5 (±2.1), respectively. One hundred twenty-three (72%) and 100 (59%) of the patients were assessed as having moderate and severe malnutrition by the GLIM criteria.

In terms of underlying diseases, malignancy was the most commonly seen in 90 (53%), followed by diabetes mellitus in 50 (29%). The mean CCI score was 3.7 ± 2.5. *Candida* spp. Isolated was 176 isolates from 170 patients. *Candida albicans* was most frequently seen in 78 (46%), followed by *C. parapsilosis* in 43 (25%). The mean EQUAR Candida scores in the patients with and without CVC were 13.7 and 11.7, respectively.

Regarding clinical course and outcomes, the median duration from hospital admission to the diagnosis of candidemia was 22 days (range 0–197). Echinocandin was the most frequently used in 118 (69%) as the initial antifungal therapy. The 30-day and in-hospital mortality were 35% and 44%, respectively.

### 3.1. Receiver Operating Characteristic (ROC) Curves of CONUT Score for 30-Day

Area under the Receiver operating characteristic (AUROC) of CONUT score for 30-day mortality was 0.723 [95% confidence interval (CI) 0.645–0.801, *p* < 0.001].

### 3.2. Prognostic Accuracy of CONUT Score for 30-Day Mortality

Table 2 shows the prognostic accuracy of CONUT score for predicting 30-day mortality. The appropriate cut-off for the CONUT score was 12 and was chosen based on the Youden Index [20].

### 3.3. Comparison of Overall Survival Time Between CONUT Score ≥12 and <12

**Figure 1:**
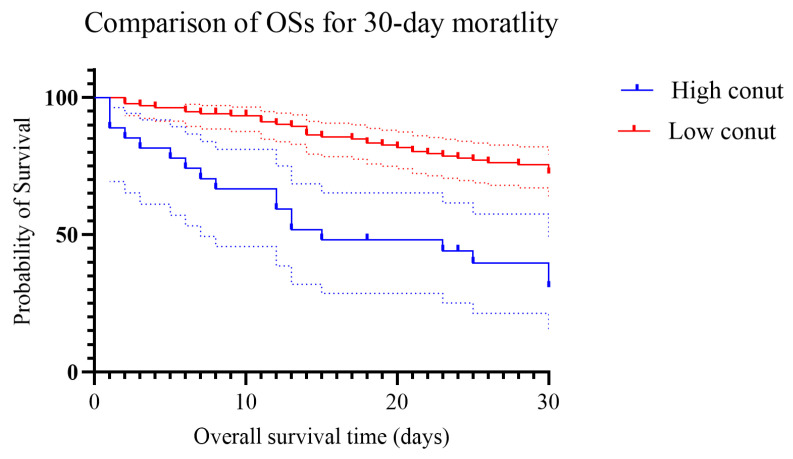
Comparison of OSs between candidemia patients with the CONUT score ≥12 and <12. Dots represent censored cases during the follow-up period.

We compared the OSs between those with the CONUT score ≥12 and <12. Patients with the CONUT score ≥12 had shorter OSs than those with the CONUT score <12 as shown in Figure 1 (*Log-Rank* test *p* < 0.001).

### 3.4. Univariate and Multivariate Analyses of 30-Day Mortality Among Candidemia Patients

Compared with the survival group, the death group had higher CONUT scores (>12), SOFA scores (>5), Charlson Comorbidity Index (CCI) scores (>3), and EQUAL Candida scores (>10), all of which were associated with 30-day mortality in patients with candidemia (Table 1). Among these variables, multivariate Cox proportional hazards analysis identified a higher CONUT score (≥12) and a higher SOFA score (≥5) as independent poor prognostic factors for 30-day mortality, whereas a higher EQUAL Candida score (≥10) was identified as an independent favorable prognostic factor (Table 3).

## 4. Discussion

In the present study, 72% of patients were classified as malnourished according to the GLIM criteria. It is reasonable that candidemia frequently develops in immunocompromised patients, regardless of the underlying cause. We found that malnutrition defined by the GLIM criteria failed to predict the 30-day mortality rate in patients with candidemia, whereas the CONUT score did so successfully. This discrepancy between the two nutritional assessment tools may be attributed to their distinct characteristics. The CONUT score includes acute inflammatory markers such as serum albumin levels and lymphocyte counts, whereas the GLIM criteria primarily reflect chronic nutritional status rather than inflammation-related changes. Because candidemia is a critical infection with a high mortality rate, chronic indicators like the GLIM criteria may not accurately assess short-term outcomes in these patients. Previous studies have also reported that the CONUT score predicted outcomes in patients with infective endocarditis [21,22] and pleural infections [23]. As these conditions similarly carry poor prognoses, the ability of CONUT to reflect inflammation-related physiological deterioration may explain its superior prognostic performance in severe infections.

The lack of prognostic significance of malnutrition defined by the GLIM criteria may be explained by several pathophysiological mechanisms specific to acute, life-threatening infections such as candidemia. The GLIM criteria were developed to diagnose malnutrition based on relatively stable phenotypic indicators (e.g., weight loss, low body mass index, and reduced muscle mass) combined with etiologic factors such as reduced intake or chronic inflammation. These components are well suited to identifying long-term nutritional deficits but are less sensitive to rapid physiological changes driven by acute systemic inflammation [24,25].

Candidemia induces a profound inflammatory response characterized by cytokine release, endothelial dysfunction, capillary leakage, and metabolic dysregulation. During this acute phase, serum protein levels, immune cell counts, and metabolic markers change rapidly, often independent of baseline nutritional reserves. For example, acute-phase reactions suppress albumin synthesis and promote lymphocyte apoptosis or redistribution, leading to transient hypoalbuminemia and lymphopenia. These alterations are strongly associated with disease severity and short-term mortality but are not captured by the GLIM framework, which intentionally excludes laboratory biomarkers to avoid confounding by inflammation [26,27]. Moreover, body weight and body mass index—key phenotypic components of GLIM—may be misleading in critically ill patients. Fluid resuscitation, edema, ascites, or third spacing frequently observed in candidemia can mask true body composition and result in inaccurate assessment of nutritional status. Similarly, the evaluation of muscle mass is often impractical or unavailable in the acute care setting, further limiting the applicability of GLIM in critically ill patients. In contrast, the CONUT score incorporates laboratory parameters that dynamically reflect acute inflammatory burden, immune competence, and metabolic reserve. Hypoalbuminemia reflects both nutritional depletion and systemic inflammation, lymphopenia indicates impaired cell-mediated immunity against Candida species, and hypocholesterolemia has been associated with adverse outcomes in sepsis due to altered lipid metabolism and impaired endotoxin neutralization. Collectively, these factors may explain why the CONUT score more accurately predicts short-term mortality in candidemia than GLIM-defined malnutrition.

There are several limitations in the study. First, this is a retrospective study in a small population. Second, those without nutritional status were excluded in this study. Then, the population never can reflect all candidemia patients’ results in the real world. There might have been a selection bias. Third, nutritional status was assessed at the time of candidemia diagnosis. Some patients may already have been affected by infection at the time of assessment. Fourth, detailed information regarding the number and duration of prior antibiotic treatments was not consistently available due to the retrospective design of this study, which may have influenced the results.

## 5. Conclusions

In conclusion, we found that CONUT score could predict the 30-day mortality in candidemia patients, while the GLIM criteria failed. The CONUT score is useful for predicting the 30-day mortality among patients with candidemia.

## Figures and Tables

**Table 1:** Comparison of patients’ characteristics and outcomes between the survival and 30-day death group (*n* = 170).

Variables	All Patients	Survival Group	30-Day Death Group (*n* = 61)	*p*-Value
	(*n* = 170)	(*n* = 109)		
Mean age (years ± SD)	73.0 ± 13.7	72.6 ± 13.7	73.7 ± 13.7	0.625
Median age (years, range)	76 (18–95)	75 (18–94)	77 (20–95)	-
Gender (*n*,%)				
Male	108 (64)	70 (64)	38 (62)	0.802
Female	62 (36)	39 (36)	23 (38)	
Nutritional status				
Body mass index (kg/m^2^) (mean ± SD)	19.2 ± 3.9	19.1 ± 4.1	19.4 ± 3.6	0.661
CONUT score	9.5 ± 2.1	8.9 ± 2.2	10.5 ± 1.6	<0.001
Prognostic Nutritional Index	26.5 ± 6.8	28.4 ± 7.0	23.1 ± 4.8	<0.001
Geriatric Nutritional Risk Index	70.0 ± 10.5	71.7 ± 11.0	66.4 ± 8.6	0.001
GLIM criteria (n,%)				
Moderate malnutrition	123 (72)	77 (71)	46 (75)	0.505
Severe malnutrition	100 (59)	63 (58)	37 (61)	0.717
Risk factors of candidemia (*n*,%)				
Prior antibiotics	148 (87)	93 (85)	55 (90)	0.367
Surgical procedure	36 (21)	26 (24)	10 (16)	0.253
Immunosuppressive agents	34 (20)	17 (16)	17 (28)	0.055
Tube feeding	39 (23)	27 (25)	12 (20)	0.448
Total parenteral nutrition	88 (52)	51 (47)	37 (61)	0.083
Chemotherapy	36 (21)	20 (18)	16 (26)	0.228
ICU admission	51 (30)	34 (31)	17 (28)	0.65
CVC insertion	117 (69)			
Source of infections (*n*,%)				
CRBSI	91 (53)	64 (59)	27 (44)	0.07
Unknown	71 (42)	37 (34)	34 (56)	0.006
Others	8 (5)	8 (7)	0	0.03
Severity of candidemia (Mean ± SD)				
Quick SOFA score	1.3 ± 1.0	0.2 ± 0.4	0.6 ± 0.5	<0.001
SOFA score	4.7 ± 3.3	2.5 ± 2.6	5.8 ± 3.5	0.009
SIRS score	2.1 ± 1.1	1.9 ± 1.2	2.2 ± 1.0	0.071
Condition (*n*,%)				
Septic shock	41 (24)	22 (20)	19 (31)	0.135
Disseminated intravascular coagulation	49 (29)	26 (24)	23 (33)	0.083
Underlying diseases (*n*,%)				
Heart disease	46 (27)	27 (25)	19 (31)	0.369
Chronic pulmonary disease	16 (9)	10 (9)	6 (10)	0.887
Hepatic disease	7 (4)	5 (4.6)	2 (3)	0.68
Diabetes mellitus	50 (29)	35 (32)	15 (25)	0.302
Chronic kidney disease	23 (14)	13 (12)	10 (16)	0.414
Hemodialysis	11 (6)	5 (4.6)	6 (10)	0.182
Gastrointestinal disease	4 (14)	3 (2.8)	1 (1.6)	0.646
Collagen vascular disease	14 (2.4)	8 (7)	6 (10)	0.57
Cerebrovascular disease	40 (24)	27 (25)	13 (21)	0.65
Malignancy	90 (53)	56 (51)	34 (56)	0.585
Metastasis	47 (28)	28 (26)	19 (31)	0.445
Paralysis	7 (4)	5 (5)	2 (3)	0.706
Charlson comorbidity index (mean ± SD)	3.7 ± 2.5	3.5 ± 2.6	4.1 ± 2.4	0.124
Charlson comorbidity index ≥ 3 (n,%)	99 (58)	58 (53)	41 (67)	0.066
Initial antifungal therapy (*n*,%)				
Echinocandin	118 (69)	82 (48)	36 (59)	0.028
Azole	17 (10)	12 (11)	5 (8)	0.545
Polyene antimycotic	22 (13)	15 (14)	7 (11)	0.877
None	13 (8)	0	13 (21)	<0.001
EQUAR Candida score (mean ± SD)				
All cases (*n* = 170)	12.4 ± 2.8	14.2 ± 2.4	11.2 ± 2.9	<0.001
With CVC (*n* = 117)	13.7 ± 3.0	14.9 ± 2.6	11.8 ± 3.1	<0.001
Without CVC (*n* = 53)	11.5 ± 2.2	12.5 ± 1.9	9.6 ± 1.5	<0.001
Duration of				
hospital stay (mean days ± SD)	68.9 ± 58.6	75.8 ± 63.6	56.4 ± 46.4	0.024
antifungal treatment (mean days ± SD)	29.5 ± 69.3	40.0 ± 84.6	10.9 ± 9.4	0.001
Outcome				
Mortality (*n*,%)				
30-day mortality	61 (35)	-	-	-
In-hospital mortality	75 (44)		-	
Pathogen isolated by blood culture (*n*,%)				
*Candida albicans*	78 (46)	45 (41)	33 (54)	0.108
*Candida parapsilosis*	43 (25)	31 (28)	12 (20)	0.207
*Candida glabrata*	35 (21)	24 (22)	11 (18)	0.538
*Candida tropicalis*	9 (5)	6 (5.5)	3 (5)	0.87
*Candida guilliermondii*	8 (4.7)	7 (6)	1 (1.6)	0.158
*Candida krusei*	1 (0.6)	0	1 (1.6)	0.18
Others	1 (0.6)	1 (0.9)	0	0.453

CONUT, Controlled Nutritional Status; CRBSI, catheter related blood stream infection; CVC, central venous catheter; EQUAL, European Confederation of Medical Mycology Quality of Clinical Candidemia Management; GLIM, Global Leadership Initiative on Malnutrition; ICU, intensive care unit; SD, standard deviation; SIRS, systemic inflammatory reaction syndrome; SOFA, sequential organ failure assessment.

**Table 2:** Prognostic accuracy of CONUT score for predicting 30-day mortality.

CONUT Score	Sensitivity (%)	Specificity (%)	PPV (%)	NPV (%)	Youden Index
≥8	40	91	97	89	31
≥10	50	71	71	61	21
≥12	72	73	38	92	44

CONUT, Controlled Nutritional Status; NPV, negative predictive value; PPV, positive predictive value.

**Table 3:** Univariate and multivariate analyses for 30-day mortality among candidemia patients.

Variables	HR	95% CI	*p*-Value	HR	95% CI	*p*-Value
CONUT score (≥12)	6.7	2.9–15.8	<0.001	2.1	1.1–4.0	0.025
SOFA score (≥5)	7.1	3.2–15.7	<0.001	2.6	1.4–4.8	0.002
CCI (≥3)	1.8	1.0–3.6 †	0.055	-	-	-
EQUAL Candida score (≥10)	0.0 *	0.0–0.1	<0.001	0.1 **	0.0–0.1 ***	<0.001

CCI, Charlson comorbidity index; CI, confidence interval; CONUT score, Controlled Nutritional Status; HR, hazard ratio; SOFA score; Sequential Organ Failure Assessment score. * 0.002, ** 0.07, *** 0.04–0.14, † 0.98–3.

## Data Availability

The original contributions presented in this study are included in the article and Supplementary Materials. Further inquiries can be directed to the corresponding author.

## Supplementary Materials

The following supporting information can be downloaded at: https://www.mdpi.com/article/10.3390/nu18060936/s1, Table S1: Comparison of Nutritional Indices.
