# Supplementary material for: Prognostic Impact of Nutritional Status in Patients with Candidemia
Source: Nutrients. 2026 Mar 16;18(6):936. doi: 10.3390/nu18060936 (PMC13029750; doi:10.3390/nu18060936)
Supplement: Supplementary file 1 [file nutrients-18-00936-s001.zip › nutrients-4182143-supplementary.pdf]

Supplemental Table S1. Comparison of Nutritional Indices

Index	CONUT score	Prognostic Nutritional Index	Geriatric Nutritional Risk Index	GLIM criteria
Components	Serum albumin, total lymphocyte count, total cholesterol	Serum albumin, total lymphocyte count	Serum albumin, body weight / ideal body weight	Phenotypic criteria (weight loss, low BMI, reduced muscle mass) plus Etiologic criteria (reduced intake/assimilation, inflammation)
Formula / Criteria	Each parameter is scored and summed (0–12 points)	PNI = 10 × albumin (g/dL) + 0.005 × lymphocyte count (/μL)	GNRI = 14.89 × albumin + 41.7 × (body weight / ideal body weight)	Malnutrition is diagnosed when ≥1 phenotypic + ≥1 etiologic criterion are present
Main Assessment	Nutritional status, immune function, lipid metabolism	Nutritional and immunological status	Nutritional status and body composition	Diagnosis of malnutrition

CONUT, Controlled Nutritional Status; GLIM, Global Leadership Initiative on Malnutrition.
